# Electrolyte-Sensing Transistor Decals Enabled by Ultrathin Microbial Nanocellulose

**DOI:** 10.1038/srep40867

**Published:** 2017-01-19

**Authors:** Jonathan D. Yuen, Scott A. Walper, Brian J. Melde, Michael A. Daniele, David A. Stenger

**Affiliations:** 1 Center for Bio/Molecular Science and Engineering, U.S. Naval Research Laboratory, 4555 Overlook Ave. SW, Washington D.C. 20375, USA; 2 Department of Electrical and Computer Engineering, North Carolina State University, Raleigh, NC 27695, USA; 3 Joint Department of Biomedical Engineering, North Carolina State University and University of North Carolina at Chapel Hill, Raleigh, NC, 27695, USA

## Abstract

We report an ultra-thin electronic decal that can simultaneously collect, transmit and interrogate a bio-fluid. The described technology effectively integrates a thin-film organic electrochemical transistor (sensing component) with an ultrathin microbial nanocellulose wicking membrane (sample handling component). As far as we are aware, OECTs have not been integrated in thin, permeable membrane substrates for epidermal electronics. The design of the biocompatible decal allows for the physical isolation of the electronics from the human body while enabling efficient bio-fluid delivery to the transistor *via* vertical wicking. High currents and ON-OFF ratios were achieved, with sensitivity as low as 1 mg·L^−1^.

Flexible and ultrathin substrates supporting microelectronic components have the potential to spur the development of pervasive physiological and health monitoring by providing biosensors and bioelectronics that can be seamlessly and imperceptibly integrated onto or into the human body^1^,^2^,^3^,^4^. Notable applications arising from these technologies include epidermal electronics^5^,^6^,^7^,^8^, imperceptible electronics^9^,^10^,^11^, and injectable electronics^12^,^13^.

Considerations for biocompatibility and environmental sustainability notwithstanding, notable drawbacks of current material technologies include the impermeability and hydrophobicity of the engineered plastic substrates utilized in conformal and epidermal bioelectronics. Hydrophobic substrates resist the permeation of bio-fluids containing analytes of interest, resulting in the sensor electronics needed for bio-fluid interrogation having to be in direct contact with the human body, potentially posing health hazards and opportunities for bio-fouling of the system. Thus far, few technologies to overcome this problem have been proposed. While they provide feasible solutions, the devices are either too complicated, too expensive, too thick for conformal electronics, or take too long for analyte delivery *via* lateral fluid flow.

In the aforementioned context of epidermal bioelectronics, we report a self-adhering bioelectronic decal that can collect, transmit and interrogate a bio-fluid. The device consists of a thin-film organic electrochemical transistor (OECT) fabricated on a thin (<20 μm), porous microbial nanocellulose membrane (Fig. 1a). Our technology possesses properties that help address the drawbacks listed above, *e.g.* the porous and hydrophilic nanocellulose substrate is not only permeable to liquids and gases, but it also allows the efficient vertical fluid delivery (wicking) of analytes entering the bottom surface to the sensing electronics on top, thereby reducing the required time for delivery of analytes. The substrate material is also bioinert, making it safe to adhere directly onto the human body. In addition, the fabrication of our bioelectronic decal is simply constructed, consisting of just four layers with printed active layers. Multiple devices can be fabricated on one substrate, and they can easily be diced, peeled from the backing substrate by moistening the nanocellulose sheet, and re-attached onto many desired surfaces. Furthermore, the entire thickness of the bioelectronic decal is less than 25 μm which enables conformal adherence to human skin.

We highlight two main components of our technology. The first component is that microbial nanocellulose is an ideal material because it is flexible, mechanically robust, hydrophilic, has tunable optical properties, is permeable to liquids and gases and, while chemically-inert, is biodegradable and biocompatible^14^,^15^,^16^. We have recently developed and reported a process to form wafer-sized nanocellulose laminate with controllable thickness to support the fabrication of bioelectronic devices^17^. The porous substrate differs from impermeable plastic films used in epidermal and imperceptible electronics, as it can wick biofluids secreted by the skin and transport them to the integrated sensors on top of the decal, providing for the isolation of the electronics from the human body. While porous, flexible, synthetic polymer membranes exist, they are not only expensive, but are also too thick (>100 μm) to achieve conformal contact with the skin, and so are generally limited to filtration applications^18^. Another advantage of nanocellulose is that it is amenable towards chemical modification – a variety of materials can be covalently anchored onto the cellulose polymer chain, such as enzymes, nanoparticles^19^, and electrochemically-active mediators, including ferrocene^20^ and boronic acid^21^. Functionalization of nanocellulose has the potential of expanding the range variety of analytes that can be electrochemically detected with similar bioelectronic decals.

The second point of emphasis is that utilizing OECT technology for our bioelectronic decal has simplified fabrication and operation. Multiple devices can be fabricated on one substrate (Fig. 1b) and they can easily be diced, peeled from the backing substrate simply by moistening the nanocellulose sheet, and re-attached onto any surface, as shown in Fig. 1c and d. Many devices have been explored for epidermal biosensing^22^,^23^,^24^, but OECTs have made extensive inroads in bioelectronics as they can interface directly with aqueous electrolytes to sense chemical and biological components resulting from redox processes^25^,^26^. Prior reports have demonstrated that OECTs, based on a doped-semiconducting polymer, poly(3,4-ethylenedioxythiophene):polystyrenesulfonate (PEDOT:PSS), can detect biological analytes such as glucose, lactose, neurotransmitters and DNA^27^,^28^,^29^,^30^. OECTs have also been successfully fabricated onto paper substrates, but as of now, typically photopaper or other types of thick paper treated with polymer coatings to achieve a smooth, impermeable surface have been used^31^,^32^,^33^. To the best of our knowledge, OECTs have not been integrated into microscale substrates for epidermal electronics. We also note that our technology is similar to paper-based analytical devices (μPADS) and lateral flow assays (LFAs)^34^,^35^. However, device based on these technologies are typically millimeters-thick and mainly rely on lateral wicking across the substrate area for analyte delivery, which take more time than direct vertical wicking. Slow response time can be a disadvantage for devices that track physiological responses which may change quickly or would require larger samples volumes to be transported for sensing. Our bioelectronic decal relies on vertical fluid delivery (wicking) of sweat across the smallest dimension of the substrate directly to the the sensing elements on top.

As a proof-of-concept for this OECT decal strategy, we designed our bioelectronic decal with the simplest type of OECT used for detection, one that monitors cation concentration in a biofluid. Under the correct bias and in the presence of cations (such as Na^+^, K^+^ and Ca^2+^), the de-doping of metallic PEDOT:PSS follows as:


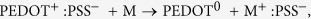


whereupon oxidized PEDOT is reduced to insulating neutral PEDOT, with a resultant drop on conductivity^36^. This change in conductivity can be used in the detection of electrolytes in biofluid, which sensors are relevant to some medical issues^37^,^38^,^39^, such as cystic fibrosis, which manifests and is monitored through a Cl^−^ ion imbalance in sweat. For the purpose of these evaluations, we utilize our technology to monitor Na^+^ concentrations. Na^+^ imbalance in sweat can be generally related to dehydration during both acute and chronic exertion. Na^+^ concentration depends on an individual’s physiological status, varies with location on the body, and correlations have been reported between heart rate and sweat sodium concentration^40^,^41^. Consequently, Na^+^ concentration was selected as a representative and useful starting point to evaluate the OECT decal. The sensing mechanisms for the OECT decal can be expanded to other biological and chemical targets as mentioned above, which can help to increase the applicability of the presented technology.

A detailed schematic of the OECT decal is presented in Fig. 1a. The OECT decal consists of the nanocellulose substrate, 10–15 μm thick, with an isolation layer of SU-8 inkjet-printed on the surface^42^,^43^. The SU-8 layer serves to isolate the source-drain electrodes of the OECT from the nanocellulose and any contact with the analyte, which can serve as a path for leakage current between electrodes. SU-8 is suitable for flexible, thin-film devices, and has been utilized in a variety applications such as flexible antennas, microfluidics and microelectronics^42^,^43^,^44^,^45^,^46^,^47^. Due to the permeability of the nanocellulose, care must be taken to ensure that the SU-8 only partially penetrates the substrate to allow the bottom of the nanocellulose to wick fluids. This is achieved using a sufficiently thick nanocellulose sheet (>5 μm) and by printing the SU-8 layer at elevated cartridge and substrate temperatures. The SU-8 layer is printed with two windows exposing nanocellulose, with the larger square window for the gate electrode, and the smaller rectangular window for the active PEDOT:PSS layer.

All OECT contacts were formed by evaporative deposition of gold with a chromium adhesion layer. While we could conceivably contact the PEDOT:PSS directly with microprobes to make electronic measurements, we found that reproducible contact could only be achieved with contact forces that deformed or damaged the PEDOT:PSS film; thus, the deposited electrodes were used as contact pads. In fact, the chromium-gold contact pads were robust, showing little change in resistance after repeated testing and mechanical stress (see Figure S1a in the Supplementary Information). To further avoid damage by the sharp microprobe tips, silver paste was finely applied to the gold pads for physical contact with the microprobe tip during measurements. In addition, improved repeatability of electrical contact to the source-drain electrodes and PEDOT:PSS was achieved with the electrodes fabricated below the PEDOT:PSS film (see Figure S1b for a symmetric negative-positive IV sweep of the drain-source). The gate electrode differs from source-drain electrodes as it is evaporated directly onto the nanocellulose substrate at the gate window, while others are deposited onto the SU-8 isolation layer. No adhesion or operational issues were observed for the gate electrodes. PEDOT:PSS is then drop-cast onto the narrower window and across the SU-8 layer, such that it contacts the source-drain electrodes. The composed OECT decals are then baked at 135 °C. This process results in a 4-inch wafer with 48 devices (Fig. 1b), which is cut into smaller strips of around 4–6 devices, which can be subsequently laminated on the desired substrate (Fig. 1c).

As previously described, we initially found that the OECT decals can be pierced by the sharp microprobe tips, so measurement on top of a soft backing substrate, such as cotton pad or skin, proved problematic for device optimization. A detailed description of device optimization is included in the Supplementary Information. For preliminary testing, a strip of OECT decals was peeled off and laminated onto a rigid substrate, *i.e.* a glass slide, as shown in Fig. 1c and d. Due to the impermeability of the glass slide, device operation with electrolyte exposure relied on lateral wicking across the nanocellulose to deliver electrolyte to the gate electrode and PEDOT:PSS. Figure 2a illustrates the testing procedure. Using a micropipette, a simulated sweat solution consisting of a mixture of sodium chloride, disodium phosphate, urea and lactic acid is applied onto the exposed nanocellulose surface surrounding the electronic decal. A detailed recipe for the sweat simulant can be found in the Experimental Section. After 15 min, the electrolyte penetrates the nanocellulose underneath the decal, and electronic measurements are made.

We measured the electronic characteristics of the OECT decals prior to application of simulated sweat. Figure 2b shows current-voltage (I-V) characteristics of the source-drain as a function of applied gate voltage (V_G_). We observe that while current is very high, nearly 10 mA, the ohmic I-V characteristics show absolutely no variation as a function of V_G_. After application of the simulated sweat, the I-V characteristics shift with V_G_, as shown in Fig. 2c. These output I-V characteristics are those of a typical normally-ON OECT, *i.e.* with increasing V_G_ the initial ohmic current exhibits increasing saturation and decreases in magnitude. When V_G_ = 2 V, source-drain current (I_SD_) is less than 1 mA. The transfer curve of the device in Fig. 2d indicates that I_SD_, at a source-drain voltage of 0.4 V (V_SD_ = 0.4 V) and at V_G_ = 2 V, has dropped to 14 μA. This corresponds to an ON-OFF ratio of 428 (I_ON_/I_OFF_ = 428) with the gate leakage current (I_G_) remaining small throughout the operation. We note that I_G_ is close to I_SD_ at V_G_ = 2 V, indicating that if gate current can be further suppressed, it is possible for I_SD_ to be decreased further at high V_G_.

With optimization of the OECT decal completed and confirmation of quality OECT performance, we proceeded to test the OECT decal on a substrate that mimicked the conditions of sweaty skin. Cotton pads were used for the substrate, since it is sufficiently porous to hold the simulated sweat and mimicked the surface roughness of skin. Examples of devices on cotton pads are shown in Figs 3a and S2a. Unfortunately, we found that further optimization was required for high performance of the decal on a soft surface, and in the interests of future researchers in this field, we have provided a detailed description of the optimizations we performed in the SI. To summarize, we found testing the decal on flexible substrate resulted in possible cracking of the SU-8 isolation layer, resulting in current shorts between the OECT electrodes. This was resolved by increasing the thickness of SU-8 by successive printing, which resulted in an average SU-8 film thickness of 1.2 μm as determined by profilometry. While the device on the glass slide had a constrained operational volume due to the ultrathin nanocellulose membrane and limited lateral wicking, the cotton pad acted as an electrolyte reservoir, and we could visually observe that the entire decal wetted within a seconds. (Response time measurement was performed only on optimized devices, as shown in Fig. 3f.) The wetting of the entire decal resulted in a large ionic current between the gate and the PEDOT:PSS film due to the increased electrolyte. Since the gate leakage current is dependent of the surface area of PEDOT:PSS at the source-drain in contact of the electrolyte, this second issue was resolved by applying the square resistance law. As long as the ratio of the length to width is retained, we can reduce the total surface area of the PEDOT:PSS active layer without significantly changing its resistance, as shown in Fig. 3a–c.

Figure 3b,c show the optimized iteration of the OECT decals. We observed the performance of the optimized device on the cotton pad is similar to that on the glass slide, with currents reaching 4 mA. When operating on a controlled, rigid surface, the maximum leakage current was at 19 μA, but for the optimized devices on a soft surface, the leakage current was reduced, with a maximum at 6 μA. I_ON_/I_OFF_ ratio was therefore improved, increasing three orders-of-magnitude. Moreover, we note that the thickness of the nanocellulose substrate also provides for rapid vertical wicking in less than a second, compared to over ten minutes for lateral wicking, thereby substantially improving readout times for such a sensor system.

The effect of electrolyte concentration on the transfer behavior of the device was also evaluated, as shown in Fig. 3d. Deionized water and simulated sweat solutions with NaCl concentrations ranging from 1 mg·L^−1^ to 20 g·L^−1^ were tested. We found that the device was responsive to all the concentrations of NaCl solution tested, with ON-OFF ratio increasing with NaCl concentration. The detection limit of the OECT decal occurs when the gate leakage current is higher than the source-drain current, which we can observe for the transfer curve at 20 g·L^−1^. For V_G_ > 1 V, the OFF region shows a weak increasing dependence that scales directly with leakage current. This indicates that the OECT decal is sensitive to NaCl concentrations as low as 17.1 μM to as high as 342 mM, representing a concentration range of 2 × 10^4^. The gate dependence observed from Deionized water is attributed to any residual salts from growth and processing of the nanocellulose. Figure 3e illustrates the measurement range of the OECT decal falls well within the concentration range of NaCl in human sweat. A comparison between the reaction times for lateral wicking and vertical wicking was also performed, as shown in Fig. 3f. At V_SD_ = 0.5 V and V_SD_ = 2 V, where PEDOT:PSS will be de-doped, we observe that it takes less than 30 s for current to drop to a minimum for a device on a cotton pad, compared to 40 min for one on a glass slide, indicating the efficacy of vertical transport in terms of fluid delivery.

The OECT decals were also tested on chicken skin moistened with simulated sweat to gauge the robustness of device performance on a biological tissue with rough, localized contours, as shown in Fig. 4a. Due to the large curvature of the chicken leg, it was very difficult to make electronic measurements directly on the leg itself, as the high applied pressure of the microprobe probes resulted in the probes either falling off the contacts or delaminating contact pads; hence, the chicken skin was peeled off and attached onto a glass slide before for evaluation. Transistor performance was comparable to the other devices tested, with currents in the milliamperes and I_ON_/I_OFF_ ratios approaching three orders-of-magnitude, as shown in Fig. 4c,d. The uniform performance of the OECT decals on various surfaces indicates the robustness, flexibility and stability of the OECT decals and bodes well for future biomedical applications.

Concerning robustness of the OECT decal design, a brief discussion on the repeatability of the microfabrication of OECT decals is also warranted. Representative data is presented and data was collected from 4–6 working devices per wafer, where each wafer was processed from different batches. Within each wafer, device repeatability is dependent on the printing quality and the mask alignment during electrode evaporation, where leakage current determines what is considered a device failure. Device yield varies between rows on the wafer—an acceptable row has a device yield of around 80%. We believe the device yield can be easily and substantially improved with optimization of printing and ink parameters.

In conclusion, we have fabricated a flexible, thin-film bioelectronic decal to be adhered onto the skin for the analysis of sweat. The decal consists of an electrolyte-sensing, PEDOT:PSS-based OECT fabricated on the top surface of a thin-film of nanocellulose, where an inkjet-printed layer of SU-8 isolates the source-drain contacts. The porosity of the nanocellulose enables the vertical wicking of fluid from a biofluid source to the active area of the OECT device, such as sweat or salivary glands. We show that the decal can perform *via* lateral wicking from the sides or *via* direct vertical wicking, enabling optimization of sample collection in an epidermal application. We show excellent OECT performance with this architecture, and the devices exhibit good saturation output behavior in the milliamperes at low voltages and excellent I_ON_/I_OFF_ ratios. Device sensitivity to NaCl concentration was as low as 17.1 μM, ranging up nearly four orders-of-magnitude to 342 mM. We believe that our electronic decal has excellent potential for applications in both epidermal and transient electronics, and future validation will include hardware optimization and human subject testing.

## Experimental

### Nanocellulose Growth and Sheet Formation

*Gluconacetobacter xylinus* cultures were continuously maintained in HS medium^48^ as static cultures at 30 °C. Mother cultures were maintained in 50 mL conical tubes containing 15 mL of sterile medium. These cultures were left undisturbed for 10–14 days to allow the culture to develop. To generate the inoculum, the pellicle was dislodged and cells dispersed using an IKA vortex. The culture was vortexed at maximum speed two times for 30 s. The pellicle and any diffuse cellulose material precipitated to the bottom third of the conical tube. Using a sterile pipette, suspended bacteria were transferred to fresh 30 mL HS medium in sterile 100 mm crystallization dishes that had previously been covered with aluminum foil and autoclaved. The inoculated crystallization dishes were covered again with the original aluminum foil and transferred to the incubator where they were incubated undisturbed for 7 days. Following this incubation, 50 mL of fresh HS medium was gently added to the surface of the basal pellicle. The cultures were returned to the incubator for 1 week to allow a second pellicle to form. The feeding process was repeated at 1 week intervals for a total of 6 weeks to form five uniform pellicles plus the initial basal pellicle.

Harvested nanocellulose pellicles were incubated at 95 °C in 0.5 M NaOH for 1 hour to denature contaminating proteins and bacteria. The pellicles were transferred to 13 × 9 in Pyrex vessels where they were washed extensively in water over a 48 hour period. Water washes were continued until pH measurements indicated the removal of base. Pellicles were stored in water containing 0.02% sodium azide to inhibit bacterial growth. To form the sheets, pellicles were spread across 10 mm glass wafers and manually smoothened to minimize air bubbles trapped between pellicle and wafer.

### Nitrogen Adsorption Characterization

Nitrogen adsorption experiments were performed on a Micromeritics TriStar II Plus at 77 K (Micromeritics Instrument Corporation, Norcross, GA). Samples were dried on a Micromeritics VacPrep 061 sample degas system at 95 °C under flowing N_2_ gas prior to analysis. Surface area was determined by use of the Brunauer-Emmett-Teller (BET) method and total pore volume was determined by the single point method at relative pressure (*P/P*_*0*_) of 0.97. An individual sheet was determined to have a BET surface area of 51 m^2^·g^−1^ and a single point total pore volume of 0.188 cm^3^·g^−1^.

### Device Fabrication

Inkjet printing of the SU-8 isolation layer was performed with a FujiFilm Dimatix DMP-2831 Materials Printer. The ink used was 40% vol. of SU-8 2 (Microchem, Inc.) in cyclopentanone which was filtered into a DMC-11610 cartridge (10 pL drop-size) with a 0.2 μm Nalgene PTFE syringe filter. During printing, the platen temperature was set at 60 °C and the cartridge temperature was set at 70 °C. The temperatures are necessary to ensure that the ink dries out before completely penetrating the substrate. Printing was performed at a resolution of 1270 DPI, with the jetting voltage range between 15–22 V and only 3 of the 16 jets used. After printing, the substrate was left to dry on the platen for about 2 hours at 60 °C, and then exposed to UV source (UVO Cleaner Model 42, Jelight, Inc.) for 1–2 min. The SU-8 isolation layer is a rectangle of 12 × 10 mm, with a square gate window of exposed nanocellulose of 8 × 8 mm and a rectangular exposed strip of 4 × 1.25 mm or 1 × 0.3125 mm for the PEDOT:PSS layer. Gate, source and drain electrode contacts (5 nm Cr and 45 nm Au) were defined though a shadow-masked electron-beam evaporation, with the source-drain electrodes each 0.75 mm away from the strip window. PEDOT:PSS solution (Clevios P, Heraeus) was drop-cast from a micropipette calibrated at 2 μL for the smaller window. The SU-8 layer is sufficiently hydrophobic to confine the PEDOT:PSS solution in the strip, but not enough to prevent fluid contact to the PEDOT:PSS solution on the source-drain electrodes. The PEDOT:PSS layer is allowed to dry for a few hours to overnight and then baked at 130–140 °C for 10 min. Silver paste is then applied with a syringe to all the electrodes. This procedure helps protect the sample from being damaged by the sharp tips of the microprobe needles, and will not be necessary for industrial applications. The completed wafer was cut into smaller decals with a razor blade, the cut pieces moistened with deionized water, and, after a minute wait, peeled off the glass wafer with a pair of tweezers. The peeled off decals were then re-laminated either on microscope slides or moistened cotton pads and left to dry.

Prior to testing, the electrodes of the re-laminated samples were coated with silver paste, and the devices dried on a hotplate at 100 °C for 10 min to ensure almost all moisture and solvent are removed. Transport measurements were performed in air using an Agilent 4550 Semiconductor Parametric Analyzer controlled by a custom LabVIEW program on a microprobe station. For each device, an initial measurement was performed without electrolyte as a control. After all devices on one substrate were measured, the electrolyte, formulated to simulate sweat was applied. The sweat electrolyte used contained 0.5 w/v% NaCl, 0.50 w/v%, Na_2_HPO_4_ 0.1 w/v% urea, and 0.1 w/v% lactic acid. The pH was adjusted to 5.5 by NH_4_OH. For the devices on microscope slides, electrolyte was applied to surround the devices, with the SU-8 isolation layer preventing the electrolyte from directly coming in contact with the electrodes and the PEDOT:PSS layer. The electrolyte will slowly wick across the nanocellulose substrate underneath the SU-8 layer and reach the underside of the gate electrode and the PEDOT:PSS layer in 10–20 min before measurements can be taken. For the devices on the cotton pad, when the electrolyte is applied on the pad, it immediately wicks and spreads throughout in a matter of seconds; the bottom of the electronic decal becomes quickly soaked and can be tested immediately.

## Additional Information

**How to cite this article**: Yuen, J. D. *et al*. Electrolyte-Sensing Transistor Decals Enabled by Ultrathin Microbial Nanocellulose. *Sci. Rep.* **7**, 40867; doi: 10.1038/srep40867 (2017).

**Publisher's note:** Springer Nature remains neutral with regard to jurisdictional claims in published maps and institutional affiliations.

## Supplementary Material

Supplementary Information

## Figures and Tables

**Figure 1:**
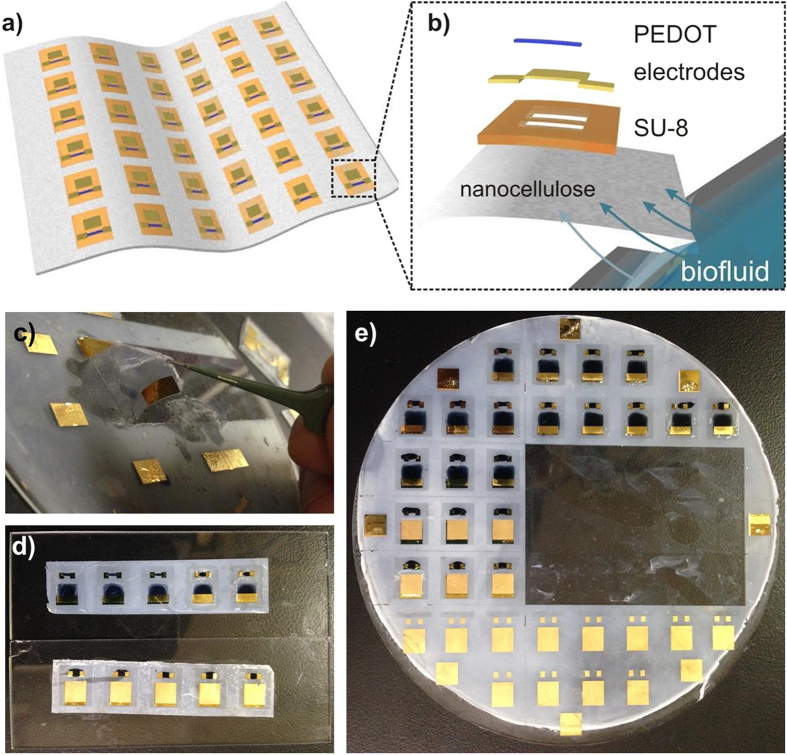
Bioelectronic decal and lamination. (**a**) Illustration of bioelectronic decal composed of a sheet of nanocellulose containing multiple OECTs; (**b**) expanded schematic of a single OECT decal, consisting of the nanocellulose substrate, an inkjet-printed SU-8 layer, evaporated gold electrodes and the drop-cast PEDOT:PSS layer. Biofluid, *e.g.* sweat, will be vertically wicked within the decal, due to the porous and hydrophilic nanocellulose substrate. The biofluid will be transported to the gate electrodes and PEDOT:PSS layer on top. (**c–e**) Representative photographs OECT decals. (**c**) Removal of the decal from the backing glass wafer after fabrication. (**d**) Laminated OECT decals on glass and (**e**) the original wafer they were peeled from.

**Figure 2:**
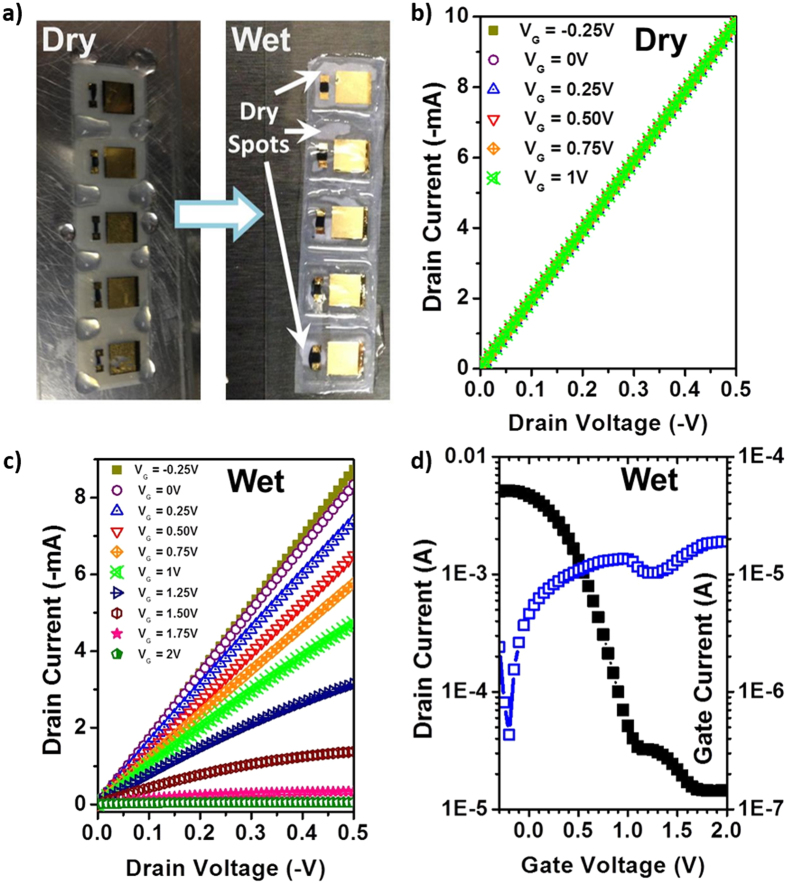
(**a**) Representative photograph of OECT decals wicking biofluid into porous nanocellulose substrate. The electrolyte thereby forms an ionic contact between the PEDOT:PSS layer and the gate electrode. (**b**) Output characteristics of the OECT decal without electrolyte (dry), but with applied V_G_, indicating that without electrolyte, there is no transistor action. (**c,d**) Output and transfer curves, respectively, of the electronic decal after application of electrolyte (wet). Normal OECT characteristics are observed, with good I_ON_/I_OFF_ ratio. For the transfer measurements, V_SD_ = −0.5 V.

**Figure 3:**
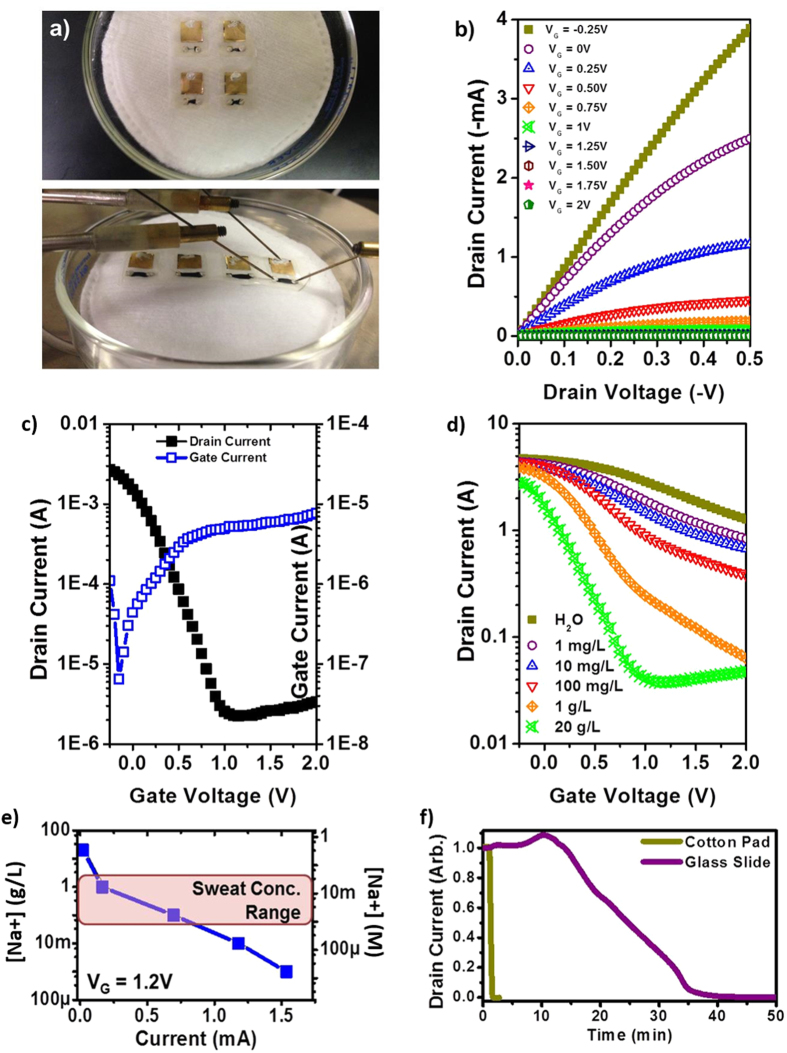
(**a–f**) The OECT decal, with optimized PEDOT:PSS active area and SU-8 isolation layer, on a cotton pad and electronic performance. (**a**) The OECT decallaminated onto an absorbent cotton pad containing a biofluid (simulated sweat). (**b,c**) Output and transfer curves, respectively, of the OECT decal after exposure to simulated sweat. Normal OECT characteristics are observed, with improved I_ON_/I_OFF_. (**d**) Dependence of the transfer curve on varied concentrations of NaCl in the simulated sweat. For the transfer measurements, V_SD_ = -0.5 V. (**e**) Current output as a function of NaCl concentration at V_G_ = 1.2 V. The highlighted region shows the physiologically relevant range of NaCl in human sweat. (**f**) Reaction times *via* normalized drain current modulation, at V_SD_ = 0.5 V and VSD = 2 V, for OECT decal on a glass slide versus on a cotton pad.

**Figure 4:**
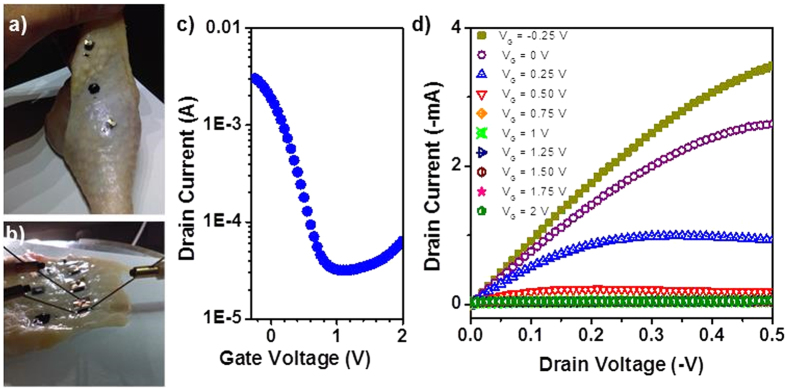
(**a–d**) OECT decal laminated to biological tissue, *i.e.* chicken skin, and resultant transistor performance (**a,b**) OECT decals on chicken skin illustrating conformal adhesion. (**c–d**) Transfer and output curves, respectively, of the electronic decal on chicken skin and exposed to simulated sweat. OECT characteristics are observed with drain currents in the mA range and I_ON_/I_OFF_ ratio of 200.
