# Supplementary material for: Electrolyte-Sensing Transistor Decals Enabled by Ultrathin Microbial Nanocellulose
Source: Sci Rep. 2017 Jan 19;7:40867. doi: 10.1038/srep40867 (PMC5244378; doi:10.1038/srep40867)
Supplement: Supplementary Information [file srep40867-s1.pdf]

Supplementary Information

Electrolyte-Sensing Transistor Decals Enabled by Ultrathin Microbial Nanocellulose

By Jonathan D. Yuen,[1] Scott A. Walper,[1] Brian J. Melde,[1] Michael A. Daniele[2, 3]* and David A. Stenger[1]

1. Center for Bio/Molecular Science and Engineering, U.S. Naval Research Laboratory, 4555 Overlook Ave. SW, Washington D.C. 20375, USA
2. Department of Electrical and Computer Engineering, North Carolina State University, Raleigh, NC 27695, USA
3. Joint Department of Biomedical Engineering, North Carolina State University and University of North Carolina at Chapel Hill, Raleigh, NC 27695

*Author to whom correspondence should be addressed; e-mail: mdaniel6@ncsu.edu

Optimization of the Bioelectronic Decals on Soft Substrate

We found that transferring the decal from a hard impermeable substrate, such as a glass slide, to a soft permeable substrate, such as a cotton pad, required further device optimization. As shown in **Figure S2a**, the initial devices tested on the cotton pad, which were from the same batch tested on the glass slide, performed poorly. In the initial testing, we observed the maximum I_{SD} of the OECT with electrolyte is over an order of magnitude lower than without electrolyte (see **Figure S2b-c)**. This result is in strong contrast to the operation of the electrolyte sensor when placed on a rigid surface, in which the decrease was less than 20%. When adhered to the soft surface, the electrolyte sensors also exhibited a linear dependence between current and V_G. This corresponds to high leakage currents near 400 μA. The high leakage current translates to equally poor OECT transfer characteristics, with an I_{ON}/I_{OFF} of 1.

We hypothesized that the combination of high I_G and low I_{SD} was a symptom of the electrolyte causing shorts among the gate, source and drain electrodes. Such shorts will contribute to increased I_G and divert current away from the drain, resulting in lower overall I_{SD}. The thinness of the isolation layer, which consists of only a single-pass printing of SU-8, coupled with nanofibrous surface topography of the nanocellulose, will result in potential susceptibility to shorting by the absorbed electrolyte. The additional strain placed on these thin SU-8 layers over a complex surface topography will either enlarge existing pinholes in the layer or cause cracking, allowing electrolyte to seep through and come in contact with the electrodes. To mitigate this problem, we strengthen the SU-8 blocking layer by increasing the thickness of the SU-8. Results are displayed in **Figure S3**. Comparing **Figure S3a** and **Figure S3b**, we conclude the maximum I_{SD} in the device with electrolyte is now commensurate to that without electrolyte, indicating no diversion of drain current. Leakage current remains high, indicating an improvement of only one order of magnitude in I_{ON}/I_{OFF} compared to the previous device, as shown in the transfer characteristic of the electrolyte-gate device in **Figure S3d**.

From this result, we hypothesized that the large leakage current related to the large surface area of the PEDOT:PSS strip in contact with the electrolyte. In the case of the decal on glass slide, the thin nanocellulose substrate and partial electrolyte coverage due to lateral wicking constrains the volume of electrolyte between the PEDOT:PSS and the gate electrode. This is not true with the cotton pad (or the skin during application), which acts as an electrolyte reservoir and wets the entire decal *via* vertical wicking, forming a high ionic current path. To reduce leakage current and optimize the device performance, we reduced the size of the PEDOT:PSS area of exposure while minimizing the resistance, to optimize performance. Since the reduction in area of a thin-film at a constant aspect ratio will result in no change in resistance, we reduced the active area of PEDOT:PSS by a factor of sixteen, as shown in **Figure 3a**. With this final modification, we improved our device performance to match that of the device tested on a rigid surface, and the results are described in the main text.

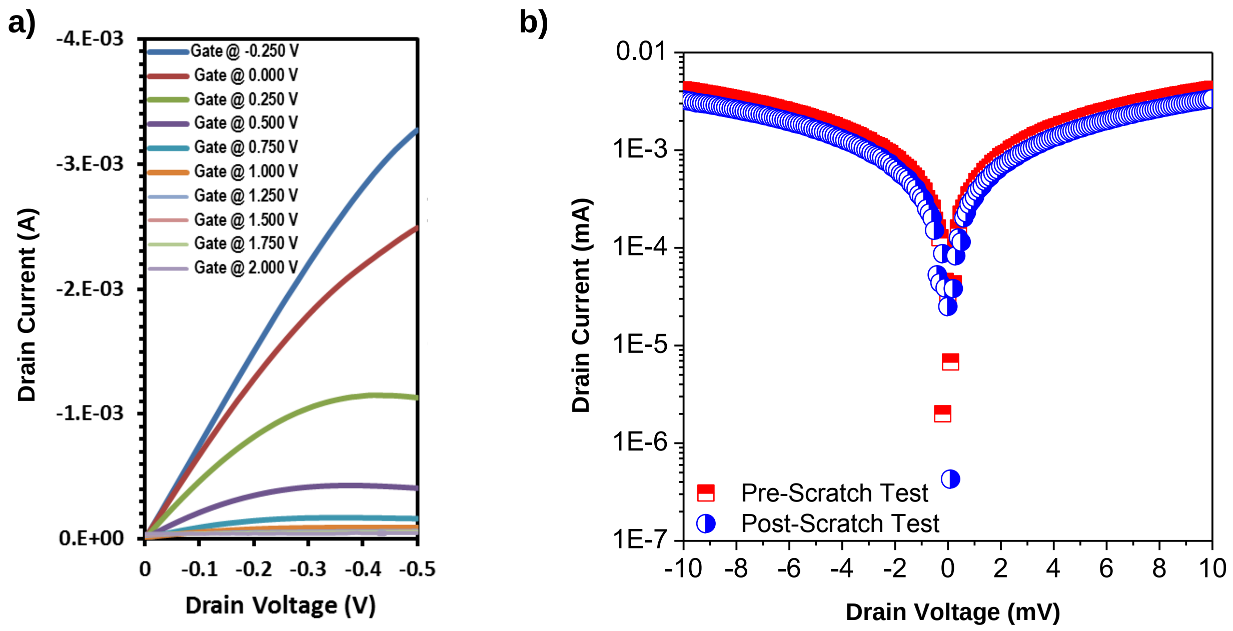

Figure S1. (a) Output curves of an OECT decal on a glass slide with silver paste as contact electrodes. Excellent OECT performance is observed in the mA range. (b) Evaluation of gold contact electrodes deposited on SU8 modified nanocellulose substrates before (red) and after (blue) scratch tests. No significant change in I-V curves and transport properties were observed. If SU8 and gold contact electrodes were brittle or poorly adhered significant degeneration of transport properties should have been observed.

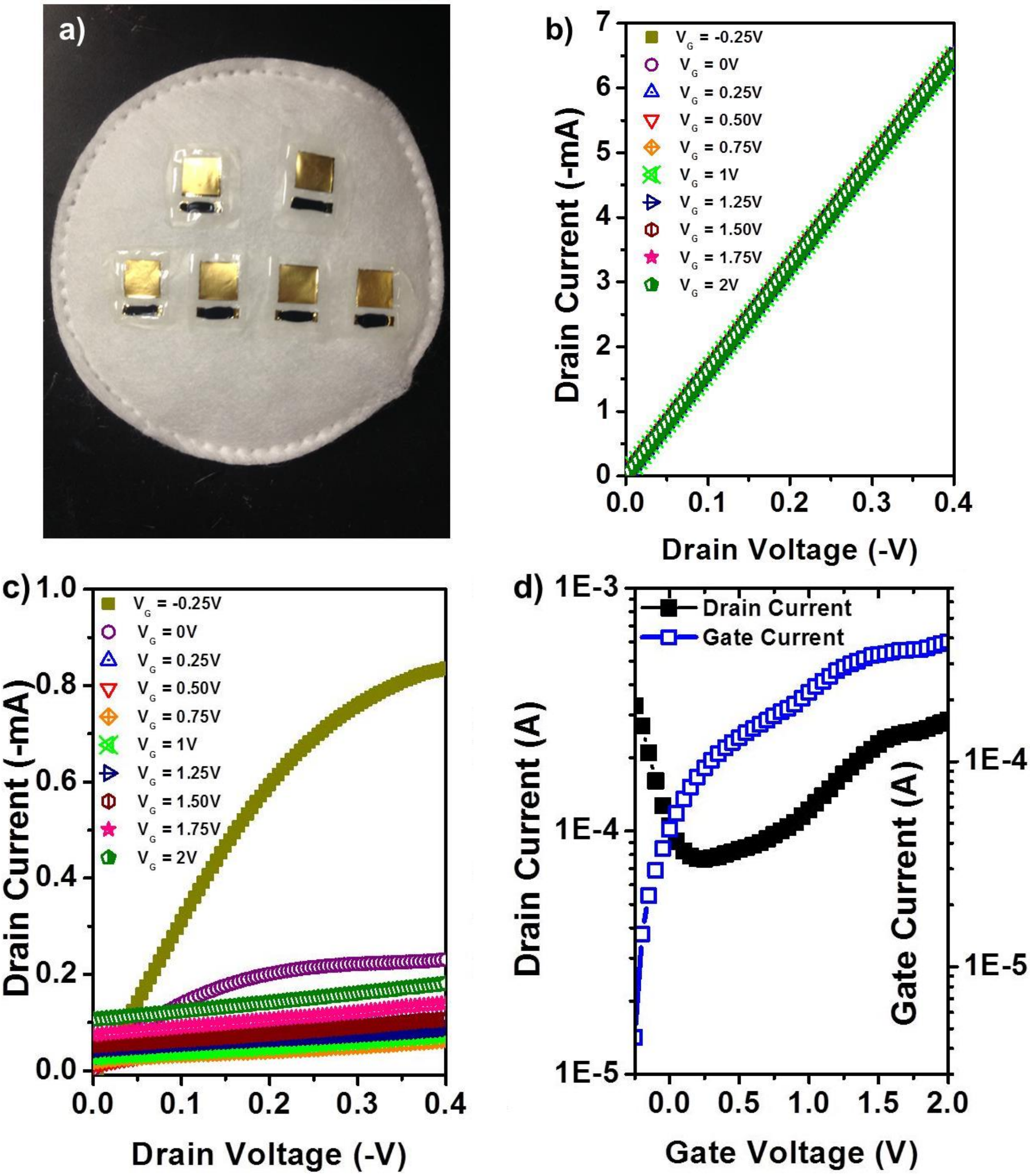

Figure S2. (a) Photograph of the OECT deal with the originally designed active area and thin isolation layer. (b-d) Transistor behavior of OECT decal. (b) Output characteristics of the decal without electrolyte and with applied V_G exhibits no transistor action. (c-d) Output and transfer curves, respectively, of the OECT decal with electrolyte introduced. Transistor characteristics are observed, but high I_G has resulted in a minima I_{ON}/I_{OFF} ratio. Reducing I_{SD} (compared to the currents observed without electrolyte) and increasing I_G results in poor output characteristics. For all transfer measurements, V_{SD} = 0.5 V.

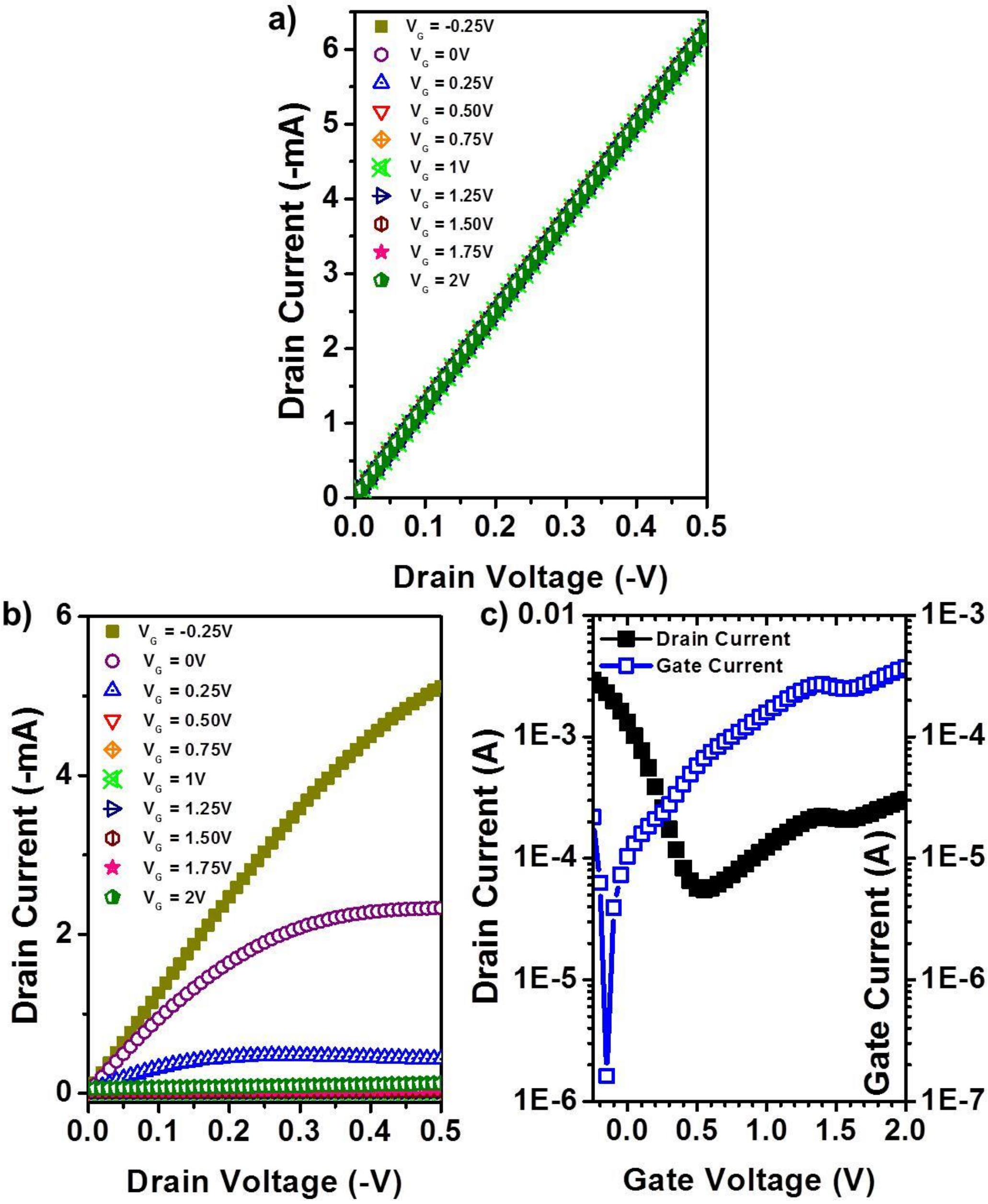

Figure S3. (a-c) Transistor behavior of the OECT decal, with its original active area and thicker isolation layer, on a cotton pad. (a) Output characteristics of the OECT decal without electrolyte and with applied V_G, indicating that without electrolyte, there is no transistor action. (b-c) Output and transfer curves, respectively, of the electronic decal after introduction of electrolyte. With reduced I_G, substantial improvement in OECT characteristics are observed. The highest I_{SD} is close to currents observed without electrolyte. However, I_G remains high and while I_{ON}/I_{OFF} ratio has improved by one order of magnitude, it is still less than the properties observed when the sample was adhered to glass. For the transfer measurements, V_{SD} = 0.5 V.
